# Effect of Robotic Delivery of Physical Activity and Fall Prevention Exercise in Older Adults: A Pilot Cohort Study

**DOI:** 10.7759/cureus.44264

**Published:** 2023-08-28

**Authors:** Christopher M Wilson, Lori Boright, Wing-Yue Geoffrey Louie, Pourya Shahverdi, Sara K Arena, Ronald Benbow, Jason R Wilson, Qinghua Chen, Katie Rousso, Nathan Huang

**Affiliations:** 1 Physical Therapy, Oakland University, Rochester, USA; 2 Physical Medicine and Rehabilitation, Corewell Health, Southfield, USA; 3 Electrical and Computer Engineering, School of Engineering and Computer Science, Oakland University, Rochester, USA; 4 Oncology and Cardiac Rehabilitation, Henry Ford Health System, Detroit, USA; 5 Human Movement Science, School of Health Sciences, Oakland University, Rochester, USA; 6 General Medicine, School of Medicine, Oakland University William Beaumont School of Medicine, Rochester, USA

**Keywords:** technology, fall prevention, exercise, robotics, physical therapy, social human-robot interaction

## Abstract

Introduction

The high prevalence of falls, lack of stability and balance, and general physical deconditioning are concerning issues for longevity and quality of life for adults aged 65 years and older. Although supervised delivery of the Otago Exercise Program (OEP) has demonstrated evidence of effectiveness in reducing fall risk of older adults, opportunities for ongoing unsupervised exercise performance are warranted. An option to facilitate exercise and performance of health behaviors may be via a social robot. The purpose of this study was to examine feasibility and initial outcomes of a robot-delivered fall prevention exercise program for community-dwelling older adults.

Methods

Five participants aged 65 years and older were recruited to receive robot-delivered modified OEP and walking program three times per week for four weeks. Outcomes of demographics, self-reported performance measures (Modified Falls Self-Efficacy Scale, Activities-specific Balance Confidence, and Almere Model assessing various constructs of acceptance of use of robotic technology), and physical performance measures (Timed Up and Go Test, Short Physical Performance Battery, Balance Tracking System [BTrackS] center of pressure sway) were collected. Data were analyzed descriptively and examined for trends in change. Measures of central tendency and distribution were used according to the distribution of the data.

Results

The mean age of the participants was 75 years (range: 66-83 years; four females and one male). The range of participant exercise session completion was 7-12 (mode=11, n=3). Constructs on the Almere Model that started and remained positive were Attitudes Toward Technology and Perceived Enjoyment with the robot. Anxiety improved from 3.80 to 4.68, while Social Presence of the robot improved from 2.80 to 3.56. The construct of Trust was somewhat negative among participants upon commencing the program and did not substantially change over time. Two participants improved their confidence on the Activities-specific Balance Confidence scale by more than 10%, while all participants showed some improvement in confidence in their balance. Mixed results were found with the Modified Falls Self-Efficacy Scale. Mean gait speed for the participants improved by 0.76 seconds over 3 meters. Improvement was also demonstrated for the Short Physical Performance Battery, with two participants improving scores by 2-3 points out of 12. No appreciable changes were found with the Timed Up and Go test and the BTrackS assessment.

Conclusion

Using a robot-led exercise program is an accessible and feasible way to deliver exercise to community-dwelling older adults in the home, but some technical constraints remain. Outcomes suggest that a four-week program is sufficient to elicit some positive trends in health outcomes and has the potential to reduce fall risk.

## Introduction

The high prevalence of falls, lack of stability and balance, and general physical deconditioning continue to be concerning issues for the longevity and quality of life of adults aged 65 years and older [1]. According to the Centers for Disease Control and Prevention (CDC), 32,000 deaths from falls occurred in 2019 alone, making it the leading cause of injury death for this age group, with a costly $50 billion in medical expenses annually [2,3]. This economic burden has been trending upward as Burns et al. reported a cost of $30.9 billion and $31.9 billion in medically treated falls in 2012 and 2015, respectively [4], and may be reflective of an aging U.S. population [5]. Insufficient physical activity and age-related comorbidities have become key contributors to the rising rates of fall-related deaths and hospital visits [1]. Because of the significance of these ailments, evidence-based balance and physical activity exercise programs have been developed, with one of the most widely accepted being the Otago Exercise Program (OEP); this program was developed to address fall risk and health issues that often come with age [6].

Although supervised delivery (e.g., by physical therapists) of the OEP has demonstrated evidence of effectiveness in reducing the fall risk of older adults [7,8], it is equally as important to consider the ongoing performance of exercise regimens when these older adults are not directly under clinician supervision. In other words, once the exercise prescription is deemed safe for the older adult to perform independently, an approach to aid in long-term exercise adherence is warranted.

One option is to facilitate exercise and the performance of health behaviors via a social robot. Although there are differences between a human and a social robot in performing physical activities, these robots still are able to elaborate on the desired motion by speaking and may be able to adequately perform and demonstrate certain exercise movements to assist older adults with their exercise activities. Social robots could potentially be an alternative means to assist with delivering therapy tasks, specifically those that demand long-term and repetitive interaction with clients of a variety of ages. For example, these robots showed their effectiveness in delivering a daily group instruction task for autism therapy for children with autism spectrum disorder [9].

Also, older adults who received cognitive interventions showed a positive attitude and acceptance toward these robots [10]. Based on a meta-analysis and systematic review by Pu et al. [11], examining older adults’ interactions with social robots, the older adults demonstrated positive trends in improved anxiety, agitation, and quality of life. Furthermore, the study found that social robots could reduce loneliness and the need for medications [11]. The evidence of the clinical benefits of exercises being delivered exclusively via robots to older adults at risk of falling is still scarce to date. Avioz-Sarig et al. [12] evaluated the perceptions of older adults when exercising along with two types of robots (e.g., NAO, a humanoid toy-like robot, and Poppy, a mechanical-like robot). Although only one session of exercise was performed, the older adults reported improved motivation to engage in exercise and reported that the robots were useful and easy to use. Spina et al. [13] compared outcomes after 20 sessions of posture and balance exercises delivered via a robotic standing platform to conventional balance exercise delivery for people with mild Parkinson’s disease (PD). The effectiveness of both exercise delivery mechanisms was found to be similar in this study. Notably, functional outcome measures were used in this study and included the Berg Balance Scale, 10-Meter Walk Test, Five Times Sit-to-Stand Test, and the Mini BESTest [13]. When considering verbal interactions and motivation, Galvão Gomes da Silva et al. [14] examined the efficacy of motivational interviewing, an evidence-based health behavior change technique, delivered by the NAO robot with older adults. In this qualitative study, most older adults reported positive interactions but felt restricted by the lack of individualized responses from the robot. This suggests that this style of personal health interaction warrants further investigation, especially in the context of exercise performance.

Despite emerging evidence examining some components of balance exercise delivery and physical activity using social robotics in an older adult population, there is a dearth of evidence that has examined the perceptions and clinical outcomes of community-dwelling older adults with a variety of comorbid health risks performing an evidence-based, widely used physical activity/exercise regimen delivered with social robots. Therefore, the purpose of this study was to examine the feasibility and initial outcomes after the development and pilot implementation of a robot-delivered exercise program for community-dwelling older adults.

## Materials and methods

Research design

After securing Oakland University Institutional Review Board (#FY2022-206) approval, a pilot cohort study of five participants aged 65 years and older was initiated. Participants were recruited via convenience sampling from the Auburn Hills Community Center in Auburn Hills, Michigan. Potential participants were informed that they would be participating in a four-week exercise program prescribed and scripted by licensed physical therapists and engineering personnel, but delivered by a robot. Additionally, the intent to obtain subjective perceptions and physical performance prior to and after one month of exercise performance was disclosed. A $10 stipend was provided for each visit attended, and the rights and privacy of the participants were protected at all times.

Inclusion and exclusion criteria

To be eligible for the study, participants had to be at least 65 years of age, ambulatory with or without an assistive device, have no self-reported diagnosis of dementia or other cognitive impairment, have no physical or medical impairment that would impact participating in standing exercises or a 15-minute walking program, pass physical and cognitive pre-screening assessments, and commit to attending at least 9 out of 12 sessions.

Participants were excluded if they had any medical or cognitive issues limiting their ability to perform safe exercises using the standardized assessments or were deemed unsafe to participate in exercises due to a physical, medical, or cognitive issue following screening by a licensed physical therapist. Participants’ abilities to participate in exercise were based on the safe exercise performance standards set forth in the American College of Sports Medicine’s (ACSM) Exercise Preparticipation Health Screening Questionnaire for Exercise Professionals [15], and, if necessary, further medical clearance. A physical therapist screening was performed by key personnel (S.A. or L.B.) to assess the ability to participate in the exercise program.

Participants were deemed clear of cognitive deficits using the Mini-Cog assessment [16] or the Trail Making Test Part B (TMT B) [17]. The Mini-Cog and TMT B were administered in person after consenting to the study as part of the prescreening assessments. If the person scored greater than 3 out of 5 on the Mini-Cog, they were not excluded. The TMT B was administered to individuals scoring three or lower on the Mini-Cog. Time greater than 273 seconds to complete the TMT B prompted exclusion from participation.

Exercise protocol

**Figure 1:**
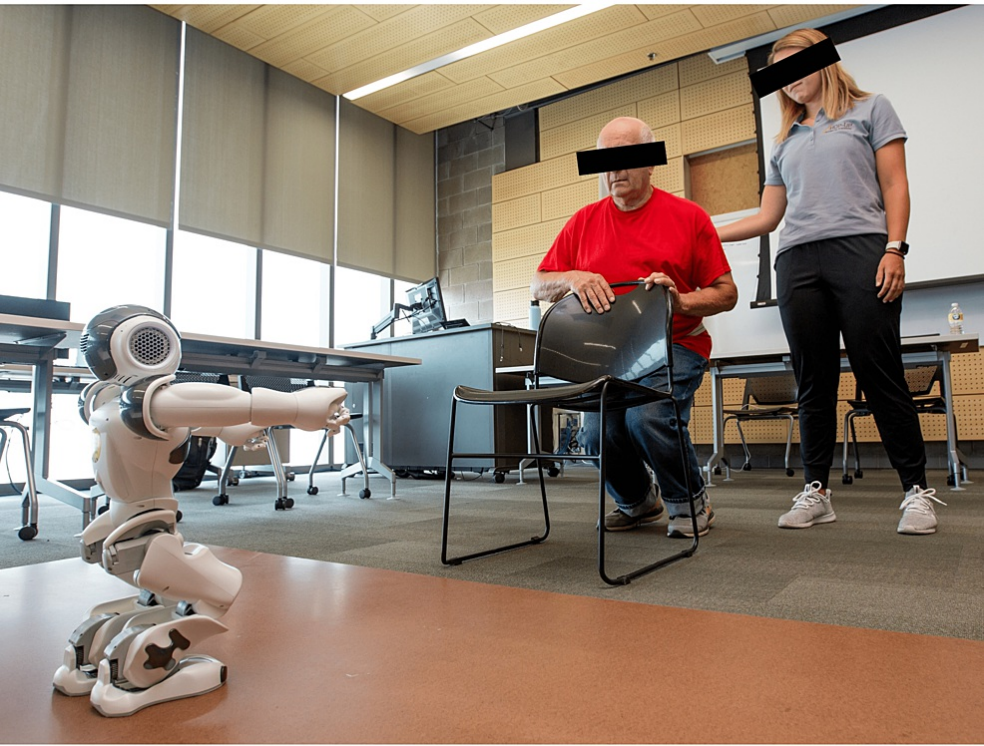
NAO robot-delivered Otago Exercise Program

**Figure 2:**
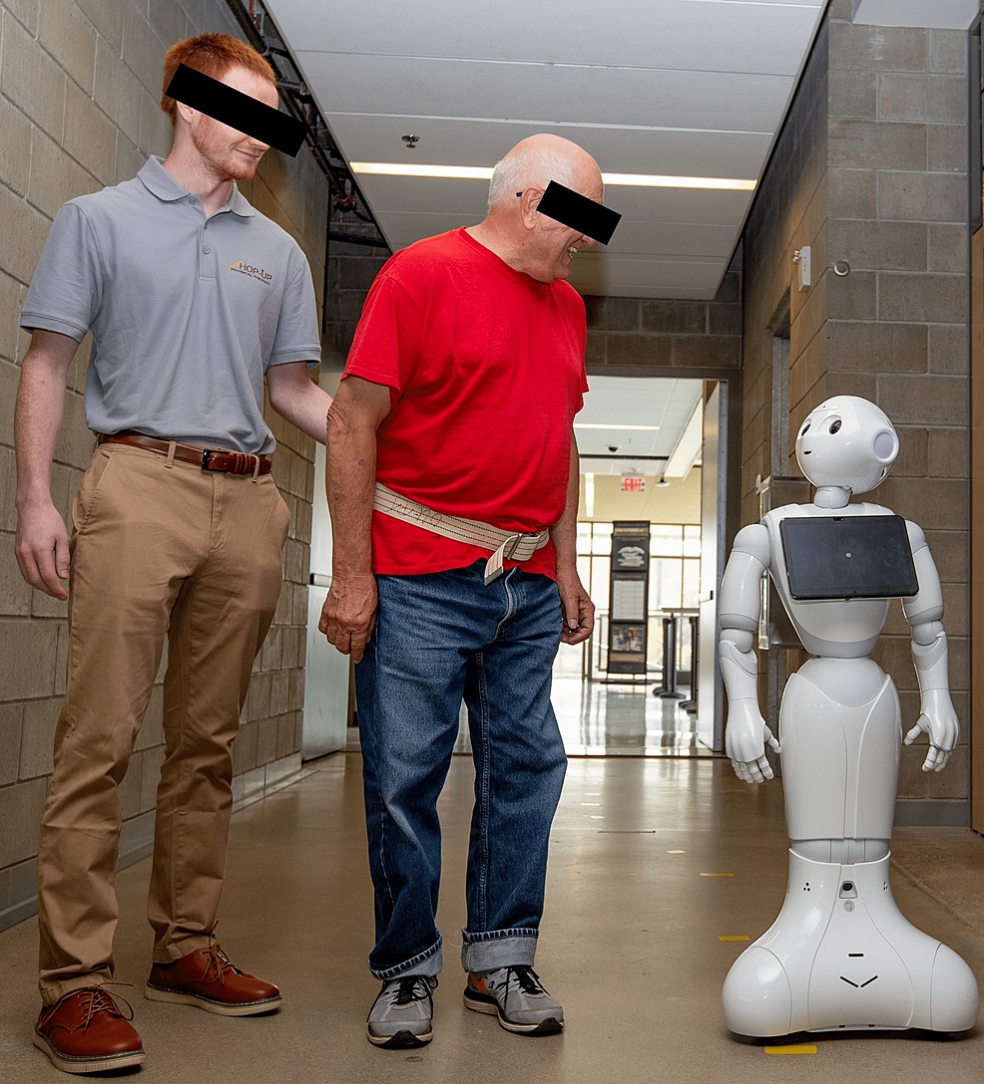
Walking program including the Pepper robot, participant, and safety personnel

The exercise interventions were delivered three times a week for four weeks and took place in August 2022 in the Engineering Center at Oakland University (OU) in Rochester, Michigan. After an initial COVID-19 and vital sign screening to assure safety to initiate exercise, a NAO social robot (Figure 1) was tasked with delivering a 15-minute modified level B OEP to the participants for the first half of the exercise session of each visit. Then, a second Pepper robot delivered a 15-minute walking program controlled via virtual reality (VR) technology by health and engineering professionals (Figure 2). It is notable that the ethics board-approved protocol required the use of a safety belt and standby human assistance during exercise interventions.

Data collection

Prior to participating in the first exercise session, baseline demographics including medical and social history, fall-related self-efficacy and confidence, social robot perceptions, and physical performance outcome measures were obtained under the supervision of licensed physical therapists. Participants returned to the research site two days after completion of the last exercise session, and the same instruments were used to gather post-participation outcomes. Table 1 summarizes the instruments and tools used during this study.

**Table 1:** Description, constructs, and measurement scale of outcome measures *Completed on visit 1 only

Outcome measure (completed pretest and posttest unless otherwise noted)	Description and construct	Measurement scale
Self-reported measures		
Demographic questionnaire*	Nine questions to establish age, sex, race/ethnicity, marital status, level of education, employment status, income, history of exercising with a robot, and prior use of technology	Categorical
Rapid Assessment of Physical Activity (RAPA) [18]*	Seven questions to briefly determine the amount and intensity of physical activity an older adult usually does.	1 = lowest level of physical activity, 7 = highest level of physical activity
Functional Comorbidity Index [19]*	Self-reported checklist of the presence or absence of 18 health conditions and was designed to predict physical functional status.	0 = lowest number of comorbidities listed, 18 = presence of all listed comorbidities
Modified Falls Efficacy Scale (MFES) [20]	A 14-activity questionnaire asking a person about their confidence in performing a variety of indoor and outdoor activities without falling. Each item is scored on a 10-point visual analog scale (0 = not confident/not sure at all, 10 = completely confident/completely sure)	0 = lowest score (less confidence and more fear of falling) 140 = highest score (more confidence and less fear of falling)
Activities-specific Balance Confidence (ABC) Scale [21]	A 16-item self-reported measure of balance confidence in various activities. Each item is rated from 0% to 100% confidence and an average confidence level is calculated.	0 = no confidence, 100 = complete confidence
Modified Almere Questionnaire [22]	29 questions assessing selected constructs of social robotic interactions (anxiety, attitude toward technology, social presence, trust, and perceived adaptiveness/enjoyment/sociability/ usefulness)	Likert scale: 1 = strongly disagree, 5 = strongly agree
Perceptions of Robotic Exercise Questionnaire	10 Likert-style questions assessing overall perceptions of exercising with a robot and perceptions of effectiveness, motivation comparing other means of exercise (group, alone, video)	Likert scale: e.g., Much less effective = lowest, much more effective = highest
Physical performance measures		
Timed Up and Go [23]	Timed assessment of a person getting up from a chair, walking 10 feet (3 meters and sitting back down)	Number of seconds
Short Physical Performance Battery [24]	Series of three tests consisting of gait speed over 10 feet (3 meters), timed repeated chair stands (5 repetitions), and static standing for 3 progressively hard balance stances	Each scale rated 0-4 (0 = lowest score, 4 = highest score). Score from each of the three tests added for a total score of 0-12.
Balance Tracking System (BTrackS) Balance Platform [25]	Balance platform measuring postural sway in static standing. Three trials of 20 seconds each.	Overall distance of postural sway measured in centimeters.

Data from participants who completed greater than or equal to half of the scheduled exercise sessions are reported in the results. The questionnaires and datasets generated during and/or analyzed during the current study are available from the corresponding author upon reasonable request.

Self-report measures

Following recruitment, interested participants were mailed a packet of pre-intervention forms to assess perceptions of exercising with robots and other lifestyle factors. Participants were instructed to bring the completed packet with them on visit 1. On visit 12, participants were asked to complete the forms once more for post-intervention data collection. Prior to program initiation, each participant completed an internally developed questionnaire on exercising with a robot, which included demographic information as well as the Rapid Assessment of Physical Activity (RAPA) [18], and the Functional Comorbidity Index (FCI) [19]. In addition, participants completed the Modified Falls Efficacy Scale (MFES) [20], a modified Almere questionnaire, and the Activities-specific Balance Confidence (ABC) scale [21,26]. See Table 1 for a description of each of the patient-reported outcome measures. With the exception of the demographic items, each participant completed each of the surveys after study participation. The Perceptions of Robotic Exercise Questionnaire was internally developed and refined after pilot testing and consisted of 10 Likert-style questions (Appendix 1). It consisted of querying the participant’s perceptions of effectiveness and motivation for exercising with a robot as compared to exercising alone, in a group, or following along with a video as well as a participant’s overall perceptions of exercising with a robot. The Almere Model questionnaire consists of 41 questions examining a wide variety of domains with acceptance of technology for older adults, including social aspects of interaction with a robot [22]. Investigators of this study selected 29 of the 41 relevant questions for participants to complete - termed the Modified Almere Questionnaire (Appendix 2).

Physical performance measures

A series of physical measures were also completed on visit 1 and again on visit 13. Physical performance measures used to gather baseline data included the Timed Up and Go (TUG), Short Physical Performance Battery (SPPB) which includes gait speed over 3 meters, the four-test balance scale, and the Five Times Sit-to-Stand Test. In addition, static balance was assessed using the Balance Tracking System (BTrackS). These tools were selected to measure participants’ baseline and post-intervention balance, ambulation, and physical performance measures. On the final visit, visit 13, participants were asked to return their completed post-intervention forms and were reassessed via the TUG, SPPB, and BTrackS.

**Figure 3:**
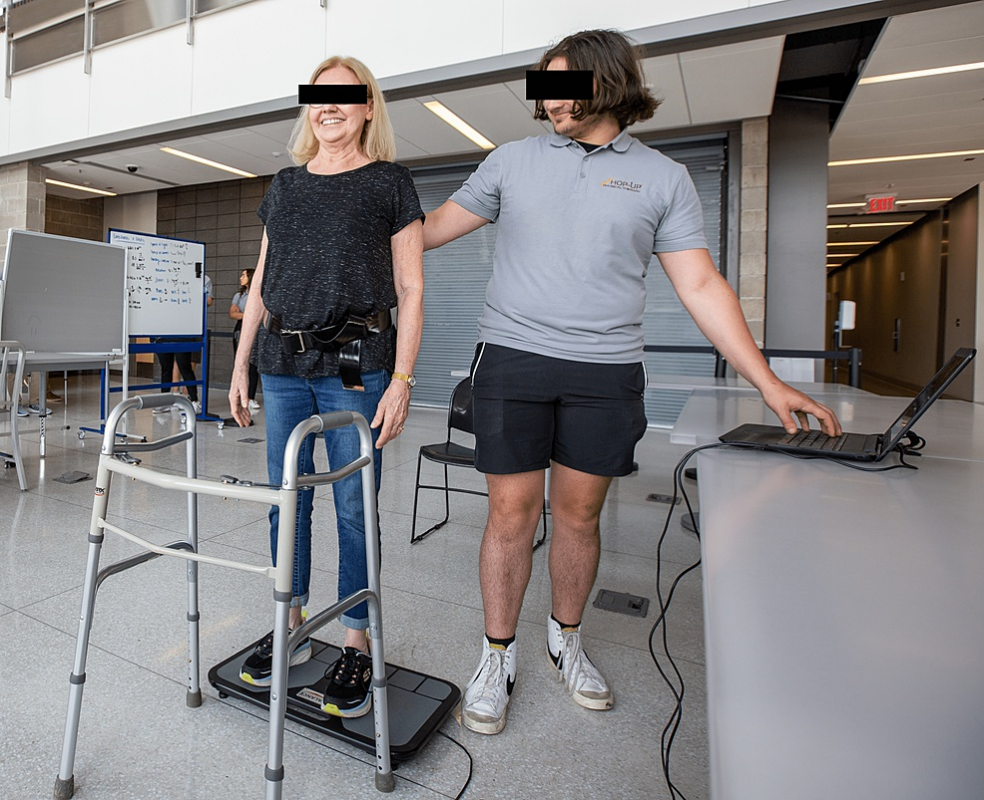
BTrackS force plate testing procedure BTrackS, Balance Tracking Systems

The TUG test is a valid, reliable measure where participants stand from a chair, walk 10 feet (3 meters), turn around, and return to sitting in the chair as quickly as possible [23]. The best of three trials was taken as the final value for the pre- and post-measurement outcomes. The SPPB is a series of three tests that has a best possible score of 12 and a lowest possible score of 0 and is a commonly used valid, reliable measure [24]. A total score is achieved by adding the scores from each of the 3 tests (0=lowest score, 4=highest score). First, participants were instructed to walk a 10-feet (3-meter) distance at a self-selected comfortable gait speed. Next, the Four Test Balance Scale was used to assess each participant’s ability to balance through a series of postures including standing with feet together, standing with the arch of one foot next to the big toe of the other, and standing tandem (heel-to-toe) for 10 seconds. Finally, the time for a person to stand from a seated position five times in a row as quickly as possible was recorded. For gait speed, the calculated score as well as the actual performance times were recorded. The BTrackS Balance Test is a clinical tool used by a variety of healthcare professionals including physical therapists to assess static standing balance by measuring the amount of sway when attempting to stand still [25]. The test was performed by having a participant stand on a force plate with their feet shoulder-width apart and eyes closed (Figure 3). Each participant was instructed to stand as still as possible for 20 seconds, while the force plate measured the excursion of the person’s center of pressure as a measure of total distance. The average of three trials was recorded.

NAO robot-delivered OTAGO exercise program

After passing the health and safety screenings, each older adult participated in the OEP Level B, which was delivered by a pre-programmed NAO social robot. The OEP consists of four levels: A (easiest) through D (most challenging). OEP Level B consists of five warm-up exercises (head, neck, back, trunk, ankle movements), three strength exercises (knee extensors, knee flexors, hip abductors), followed by eight balance exercises (knee bends, backwards walking, walking and turning around, sideways walking, tandem stance [heel toe stand], tandem walk [heel toe walk], one leg stand, and repeated sit to stand) [27]. The OEP is able to be individually tailored and is designed to be completed by older adults at least three times a week and has been demonstrated to be effective in reducing falls and increasing functional stability in older adults. One retrospective study by Knott et al. occurring over a five-year timeframe demonstrated that when comparing physical therapy interventions to the older adults’ performance of the OEP, both interventions significantly decreased the fall risk of each group’s participants [28]. The demonstration and verbal instruction of the NAO robot was scripted by the physical therapist investigators (C.W., S.A., L.B.) and further validated and refined by Doctor of Physical Therapy graduate students. Emphasis was placed on the demonstration and verbal instructions being similar to that of a physical therapist interaction. This included social conversation, education, and actual performance of the exercises.

The participants completed the modified OEP, which lasted approximately 30 minutes and consisted of the exercises described in Table 2. At the start of each intervention session, participants were screened for health status changes since the last visit. Additionally, pre- and post-exercise resting vital signs (e.g., blood pressure [BP], heart rate [HR], and oxygen saturation) were measured at each encounter to guide clinical judgment for safe initiation of exercise. A standardized seated position with feet on the floor, back supported, and measurement site at the level of the right atrium was used to measure resting BP and HR using an automated BP device (Omron HEM-712C Automatic Inflation Blood Pressure Monitor, Omron Corporation, Kyoto, Japan). The American College of Cardiology/American Heart Association BP categories of normal (less than 120 mm Hg systolic BP [SBP] and less than 80 mm Hg diastolic BP [DBP]), elevated (120-129 mm Hg SBP and less than 80 mm Hg DBP), stage 1 hypertension (130-139 mm Hg SBP or 80-89 mm Hg DBP), or stage 2 hypertension (greater than 140 mm Hg SBP or greater than 90 mm Hg DBP) were used to classify participants’ pre-exercise BP measures [29]. Vital sign measures that contraindicated exercise required participant’s exercise participation to be paused until their physician was contacted and cleared the individual to resume exercise participation.

**Table 2:** NAO robot-delivered modified Otago Exercise Program Level B Note: Otago Level B exercises are not included as they were not technically feasible for NAO to demonstrate: trunk rotation, walking in a figure 8, and heel toe standing

Exercise	Number of sets	Number of repetitions	Adaptations made
Ankle dorsiflexion (seated)	1	10x / leg	None
Long arc quads (seated)	1	10x / leg	None
Cervical neck rotations (seated)	1	5	None
Chin tucks (seated)	1	5	Demonstration of proper performance by safety personnel due to limited neck mobility of NAO
Thoracic extension (standing)	1	5	Demonstration of proper performance by safety personnel due to limited spine range of NAO
Single-leg hamstring curl (standing)	1	10x / leg	Participant holding on to chair
Hip abduction (standing)	1	10x / leg	Demonstration of proper performance by safety personnel due to NAO’s demonstration not maintaining midline in standing
Single-leg knee flexion (standing)	1	10x / leg	Participant holding on to a chair
Backwards walking	2	2 laps	NAO did not walk in a straight line backward
Sideways walking	2	2 laps	NAO did not walk in a straight line sideways
Single-leg balance	1	1x / leg	Demonstration of proper performance by safety personnel due to NAO’s demonstration not maintaining midline in standing
Sit to stand	1	5	NAO performed this more slowly than the older adults were able

The NAO robot control interface

**Figure 4:**
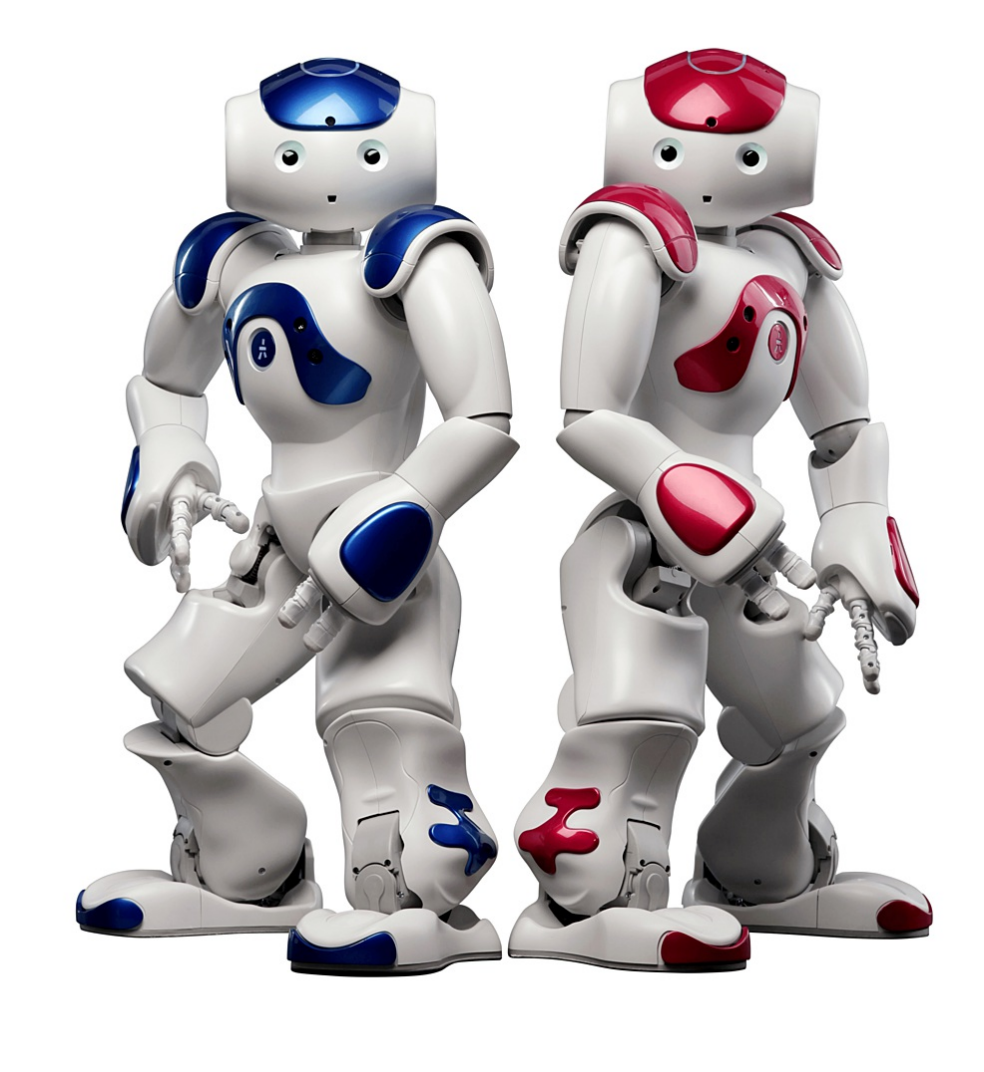
NAO robot Image created by SoftBank Robotics Europe, Inc. Own work. https://commons.wikimedia.org/wiki/File:NAO_Robot_(bleu_et_rouge)_.jpg. Licensed for reuse under Creative Commons Attribution-Share Alike 4.0 International.

**Figure 5:**
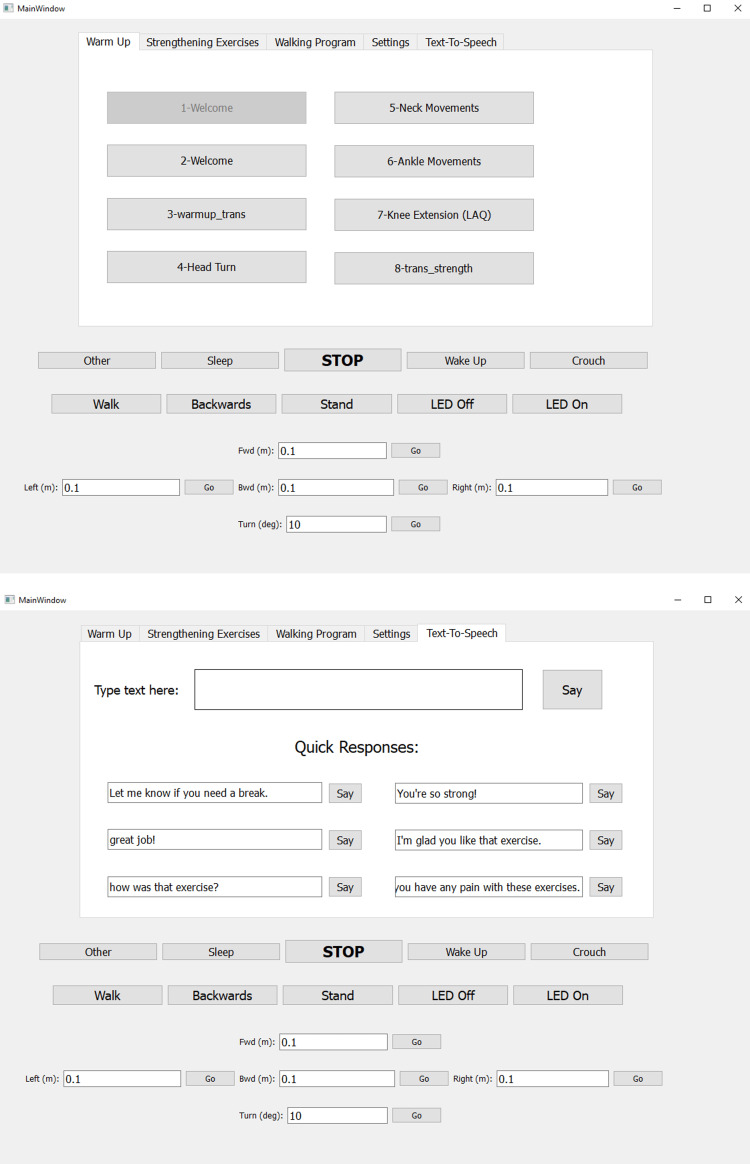
Screenshots of the graphic user interface used to control the NAO robot

The NAO social humanoid robot (Figure 4), manufactured by SoftBank Robotics, Tokyo, Japan, is primarily used for research and education purposes, but it has also been applied in a variety of other fields, such as entertainment, therapy, and customer service. NAO stands about 58 cm (23 in) tall and weighs about 5.5 kg (12 lbs). NAO Version 6 is equipped with a range of sensors, including cameras, microphones, and touch sensors, which allow it to perceive its environment and interact with people. It also has 25 degrees of freedom that allow it to move and generate a variety of different exercise motions [30]. A customized Wizard-of-Oz (WoZ) Graphical User Interface (GUI), depicted in Figure 5, was developed using PyQT and the NAOqi software development kit (SDK) from SoftBank Robotics to control the NAO robot’s behaviors. All the modified OEP Level B exercises mentioned in Table 2 were first handcrafted using the Softbank Robotics Cheorographe software’s animation mode as behaviors. Animation mode in Cheorographe enables researchers to design and record a complex motion using kinesthetic teaching [31]. Each exercise consisted of its motion behavior and verbal instructions scripted by the physical therapist investigators (C.W., S.A., L.B.) as discrete phrases that needed to be acted out sequentially. These behaviors were connected to the GUI interface and called by the robot operator during the experiment in real time. This GUI also enabled the robot operator to speak to participants using the text-to-speech functionality and modify the robot’s position with a variety of walking commands. In this experiment, the robot controller was located in the same room as the participants, but the participants were not told that the robot was controlled by direct human input.

Exercise procedures with NAO

In order to assure the safety of each participant and prevent falls, one key personnel trained in safe physical guarding during balance exercises remained within arm’s length from the participant and a safety belt was applied for all exercise performance. The key personnel were instructed to minimize interaction with the older adult in order to focus on prioritizing the robot-older adult social interaction. Two phases of the OEP Level B were integrated during these visits: warm-up and strengthening interventions (Table 2). As these exercises were performed throughout each session, modifications were made to the NAO robot’s demonstration and performance of the exercises due to the balance and range-of-motion limitations of the robot. In addition to the demonstrations by NAO, study personnel also provided verbal and manual cues to ensure the safety of the participant when the desired performance deviated from the NAO robot’s demonstrations. Key personnel were trained in how and when to intervene to assure safe proper performance of the exercises within OEP guidelines. These cues were initially provided for most exercises on the early sessions but were reduced substantially in future sessions as the participant became more familiar with proper exercise performance. For example, it was less feasible for the robot to keep up with exercises requiring single-leg balance, particularly in the later stages of each visit when the robot’s joints became overheated. It should also be noted that the NAO robot fatigue did result in the robot occasionally falling over when pushed to the extremes of its base of support. To accommodate this possible occurrence, the human assistant was instructed to step in to demonstrate the intended exercise until the NAO robot was capable of returning to the exercise leader role. Immediately before and after engaging in the OEP, participants’ perceived level of exertion was measured via the Borg Rating of Perceived Exertion Scale [32].

Walking program with the Pepper robot

**Figure 6:**
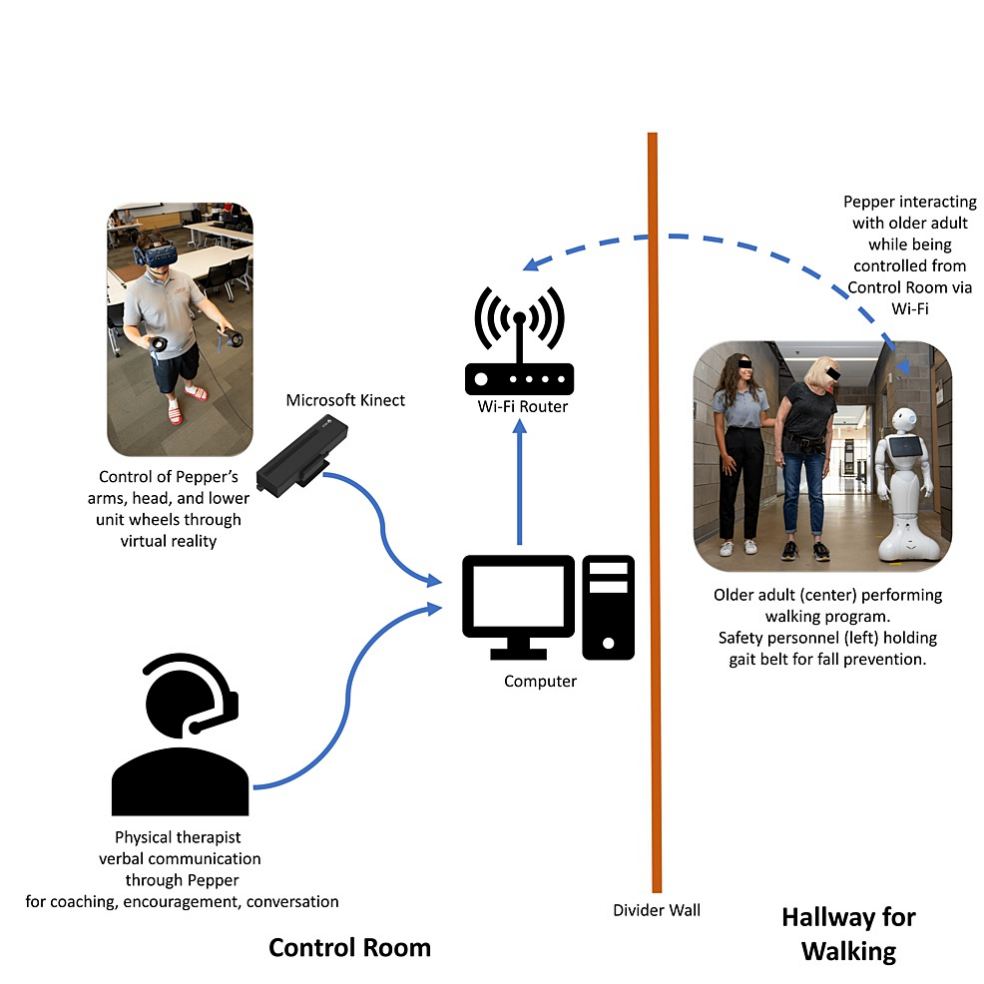
Controlling the Pepper robot A person controlling and piloting the Pepper robot via virtual reality. A second person with a headset and microphone relayed voice communications with the participant through the Pepper robot. A safety personnel with a gait belt escorted the participant during ambulation with Pepper for fall prevention. Image created by Christopher Wilson (printed with permission)

Following the performance of the modified OEP with the NAO robot, participants were escorted into a nearby walkway to perform a walking program with the Pepper robot (Figure 2). Participants were asked to complete as many laps of a 200 ft (61 m) distance as tolerated throughout a 15-minute time span and were accompanied by study personnel for safety and as required by the ethics board-approved protocol. Participants were also allowed to rest in either a seated or standing position as frequently as desired during the walking session. During the walking program, Pepper’s operators were located in a separate room using a VR headset and controllers to control the robot’s body language (head and arm movements) and verbal communication to interact with participants in real time (Figure 6) [33]. A limited amount of rolling along the hallway and Pepper’s verbal interactions with participants were controlled by the key personnel who were healthcare professionals (C.W., L.B., S.A., R.B.). Participants were asked by the operator to use the Borg Rating of Perceived Exertion Scale [32] at the halfway point (∼7 minutes) and upon completion of the walking session to assure safe exercise dosing. After each participant’s visit, a record of participant performance and other key outcomes were noted and were securely kept in each participant’s numerically assigned binder.

The Pepper robot control interface

Similar to the NAO, the Pepper is a social humanoid robot, developed by SoftBank Robotics, designed to be a friendly and interactive companion for use in homes, businesses, and public settings. Pepper, depicted in Figure 2, is equipped with a range of sensors and cameras that allow it to perceive and understand its environment, as well as to recognize and track people. It has a touchscreen on its chest that can be used to display information and interact with people.

Pepper is also equipped with a variety of actuators and motors that allow it to move its head, arms, and its wheeled base. Pepper is intended to be a social companion and can also be programmed to perform various tasks and functions, such as providing directions or assisting with home automation [34].

The Pepper robot imitated the live motion of its operator by capturing the robot operator’s joint positions in three-dimensional space using a Microsoft Kinect I and providing this data into an inverse kinematics solver [35]. While interacting with participants, the robot operators were able to view the Pepper robot’s camera using GStreamer (https://gstreamer.freedesktop.org), hear the robot’s microphone data in the VR headset, speak through the VR headset microphone and robot speakers, control the robot’s head pose using the Inertia Measurement Unit (IMU) data from the VR headset, and control the robot’s arms using the solution mentioned above. This system was implemented in Robot Operating System (ROS) Melodic Morenia 2018 (http://wiki.ros.org/melodic). The Pepper robot’s maximal rolling speed was slower than that of the participants, and thus as the participant (and the safety personnel walking with them) walked by, Pepper would move animatedly, speak encouragingly to the participant, and roll along for a short distance to maximize engagement with the robot during exercise. For the verbal interactions, the healthcare provider provided short concise words of encouragement, asking if the patient needed a rest, social conversation, or entertainment (jokes, etc.).

Data analysis

Data were analyzed descriptively and examined for trends in change. Measures of central tendency and distribution were used according to the distribution of the data. The changes in the participants’ data were evaluated for each test both individually and averaged for the group on that particular clinical assessment measure. As each component of the SPPB (gait speed, standing balance, and repeated chair stands) is routinely used alone in clinical practice, the results of each test were evaluated individually and analyzed together as the total SPPB score. Additionally, six of the 29 questions from the modified Almere Questionnaire used a 1=Strongly Disagree rating as the most positive rating, while the other 23 items had 5=Strongly Agree rating as the most positive rating. For these six items, the scoring was reversed (1=5, 4=2, etc.) in order to assess trends in mean improvement by construct category. Given the small data set anticipated for this pilot cohort study, inferential statistical testing (including statistically significant changes over time) was not conducted or reported. However, when available, changes in outcomes were compared to the minimal clinically important difference (MCID), which are scores that are commonly used by rehabilitation and healthcare professionals. MICD are scores that reflect changes in a clinical intervention that are meaningful to an individual [36]. In other words, the MCID is a meaningful measure to demonstrate improvement or regression that is clinically relevant to actual health outcomes.

## Results

Demographics and baseline characteristics

No potential participants were excluded for health, safety, or cognitive reasons. Five participants participated in the robot-led exercise sessions. One participant completed all 12 exercise sessions, three people completed 11 sessions, and one person completed seven sessions. The average age of the participants was 75 years (range: 66-83 years), with four identifying as female and one as male. Four participants reported being White or Caucasian and one as African American or Black. One participant was single, while the other four were married. All participants were retired with a variety of annual household incomes (range = US$10,001 to US$100,000). Three participants had a bachelor’s degree, while two had a high school level education. No participants reported exercising with a robot before. All five participants reported having a smartphone, while three of the five participants also reported using a tablet and a computer. One person reported using a smartwatch. Three (60%) of the participants reported a fall in the past year with one reporting an injury. See Table 3 for additional participant characteristics. 

**Table 3:** Participant characteristics RAPA scores: 2 - I do some light or moderate physical activities, but not every week; 3 - I do some light physical activity every week; 5 - I do vigorous physical activities every week, but less than 20 minutes a day or 3 days a week. Functional Comorbidity Scale range: 0 = fewest comorbidities, 18 = highest number of comorbidities. RAPA, Rapid Assessment of Physical Activity

	Participant 1	Participant 2	Participant 3	Participant 4	Participant 5
Age	83	75	66	70	81
Sex	Female	Female	Female	Female	Male
Functional comorbidity score	7	4	3	2	6
Pre-session blood pressure	182/62	142/78	122/88	123/73	136/73
Stage of hypertension	Hypertensive crisis	Hypertension stage 2	Hypertension stage 2	Elevated	Hypertension stage 1
Post-session blood pressure	156/58	155/72	124/83	138/78	157/77
Stage of hypertension	Hypertension stage 2	Hypertension stage 2	Elevated	Hypertension stage 1	Hypertension stage 2
RAPA score	5	2	3	2	2

Initial visit pre-exercise BP categories identified three participants who met the criteria of a stage 2 hypertension measure, with a stage 1 hypertension and elevated measure identified in one participant for each criterion. Notably, one participant who met the criterion of stage 2 hypertension and had SBP readings greater than 180 mm Hg that required two exercise sessions to be canceled, physician clearance obtained, and ongoing close BP monitoring. Ultimately, medication regimen adjustments were made by the participant’s physician which normalized BP to safe exercise participation ranges.

Self-report measures

Mixed results from the Modified Almere test were demonstrated and are reported in Table 4. Constructs that started positive (>4) and remained positive were Attitudes Toward Technology and Perceived Enjoyment. Anxiety improved from 3.80 to 4.68, while Social Presence improved from 2.80 to 3.56. Participants were generally neutral with regard to the robots’ Perceived Adaptiveness and Perceived Sociability as this score did not change substantially after the program. Finally, the construct of Trust was somewhat negative among participants upon commencing the program and did not substantially change over time. Participants’ responses to the Perceptions of Robotic Exercise Questionnaire (Table 5) suggested that the older adults believed exercising with a robot would be more effective and motivating than exercising alone with minimal to no change in this perception before and after participating in the program. Furthermore, participants generally agreed that exercising with a robot improved access to exercise and that they would be willing to use a robot in their home. Conversely, at program completion, participants’ responses were neutral or trended toward more disagreement with the statement that exercising with a robot was more effective or motivating than exercising with a group or with an exercise video and reflects a negative change from the program onset. Additionally, participants disagreed that exercising with a robot is preferred to exercising with another person after completing the program. Prior to starting the exercise program, the mean score of the MFES was 98.36 (100 highest self-efficacy), which decreased to 95.81 (-2.55). Notably, participant 1 had a decrease in self-efficacy of 12.86%, while the other participants remained stable. Kwok et al. [37] estimated the MCID for the MFES to be 1.5%. For the ABC scale, the mean score prior to exercising was 82.8 (100 highest balance confidence), which improved to 88 (+5.24) after the exercise sessions. The MCID for the ABC has not yet been established; however, two participants improved their confidence by more than 10%, while all participants showed some improvement in confidence in their balance. Table 6 provides details of these results.

**Table 4:** Results of the modified Almere Questionnaire by construct 1=least positive perception, 5=most positive perception

Construct (number of questions)	Mean score pre-exercise	Mean score post-exercise	Change in score
Anxiety (n=4)	3.80	4.68	1.05
Attitude towards technology (n=3)	4.00	4.13	0.13
Perceived adaptiveness (n=3)	3.47	3.60	0.13
Perceived enjoyment (n=5)	4.05	4.60	0.55
Perceived sociability (n=4)	3.55	3.50	-0.05
Perceived usefulness (n=3)	3.67	3.40	-0.27
Social presence (n=5)	2.80	3.56	0.67
Trust (n=2)	2.60	2.60	0

**Table 5:** Results from the Perceptions of Robotic Exercise Questionnaire Note: (+) Improved favorability of working with a robot, (-) Worsened favorability of working with a robot

Comparative effectiveness of exercising with a robot vs other common exercise methods		Mean score pre-exercise	Mean score post-exercise	Change
Effectiveness of exercising with a robot vs other common exercise methods (1 = much more effective, 5 = much less effective)	Alone	2.2	2.2	0
	Group	2.4	3.6	-1.2
	Video	2.4	3.4	-1
Motivation when exercising with a robot vs other common exercise methods (1 = much more effective, 5 = much less effective)	Alone	1.8	2	-0.2
	Group	2	3.2	-1.2
	Video	1.8	2.8	-1
Overall perceptions of exercising with a robot (1 = strongly agree, 5 = strongly disagree)		Mean score pre-exercise	Mean score post-exercise	Change
It will be (or is) difficult to follow along with exercises demonstrated by a robot		3	3.8	0.8 (+)
Exercising with a robot is preferred to exercising with a person		2.4	4	1.6 (-)
Exercising with a robot improves my access to exercise		2.2	2.6	0.4 (-)
I would use a robot in my home to exercise if I was provided with one		2.2	2.4	0.2 (-)

**Table 6:** Reported fall efficacy and confidence measures per participant MFES, Modified Falls Efficacy Scale; ABC, Activity Balance Confidence

Participant number	MFES			ABC		
	Pre-exercise	Post-exercise	Change	Pre-exercise	Post-exercise	Change
P1	100	87.14	-12.86	85.62	96.25	10.63
P2	99	99	1	93.75	96.25	2.5
P3	100	100	0	96.87	98.1	1.23
P4	96.4	95.5	-0.9	76	86.25	10.25
P5	96.4	96.43	0.03	82.76	88	5.24
Mean Score	98.36	95.81	-2.55	87.00	92.97	5.97

Physical performance measures

Table 7 summarizes the mean of the results of the participants’ physical performance measures pre- and post-program participation and then provides the difference between these measures.

**Table 7:** Clinical measures mean scores and differences between pre- and post-program participation TUG, timed up and go; 5xSTS, 5 x sit-to-stand; SPPB, short physical performance battery

Outcome Measure	Unit	Mean score pre-exercise	Mean score post-exercise	Difference
TUG	seconds	13.14	11.3	-1.84
Gait speed over 10 feet (3 meters)	seconds	4.08	3.32	-0.76
Gait speed	meters/second	0.74	0.91	0.17
5xSTS	seconds	14.3	15.18	0.88
SPPB	Gait speed category (0-4)	3	3.6	0.6
	Repeated chair stands category (0-4)	2.4	2.0	-0.4
	Balance category (0-4)	3.6	4	-0.4
	Total score (0-12)	8.8	9.4	0.6
BTrackS Center of Pressure Sway	cm	49.6	54.6	5

Timed Up and Go Test

The minimal clinically important improvement for the TUG in older adults ranges from 0.8 to 1.4 seconds [38]. Participants in this study improved their TUG score by 1.84 seconds, thereby exceeding the minimal clinically important improvement range.

Gait Speed

Gait speed for our participants improved by 0.76 seconds over 3 meters (0.17 meters/second). A systematic review of a variety of gait speed measures found changes in gait speed of 0.1 to 0.2 m/s to be clinically important in older adults with a variety of pathologies [39], but Kwon et al. [37] determined that 0.08 m/s was considered a “substantial change” in older adults.

Five Times Sit To Stand

Participants did not improve their Five Times Sit-to-Stand Score as the mean score prior to starting the program was 14.3 seconds and increased to 15.18 seconds, a difference of 0.88 seconds. It is notable that the slower performance by the older adults was not greater than the MCID of 2.3 seconds [40]. Participant 1 improved their score by 3.75 seconds, while participant 5 demonstrated a decline by 3.06 seconds in their performance.

Short Physical Performance Battery

Participants varied with regard to the SPPB, but an overall improvement of 0.6 was noted. Kwok et al. [37] found that a range of 0.4 to 1.5 was the MCID. Participants 1 and 2 improved their SPPB from 6 to 9 points and 8 to 10 points, respectively, indicating that these two individuals made clinically important improvements in their functional status.

BTrackS Center of Pressure Sway

Participant 4’s average postural sway improved by -13 cm, while participant 2’s postural sway worsened by 34 cm. Participants 1, 3, and 5 remained relatively stable at 2, 1, and 6 cm, respectively, which are below the threshold of the minimal detectable change score for the BTrackS, which is 9.6 cm [41].

## Discussion

This study examined the feasibility and initial outcomes after the development and pilot implementation of a robot-delivered exercise program for community-dwelling older adults. A key finding that the OEP guided by the NAO robot and a walking program delivered by the Pepper robot to older adults was indeed feasible and demonstrated positive trends among the older adult participants. This is in congruence with a prior study that concluded that the NAO robot was successfully used as a cooperative trainer for rehabilitation therapy among individuals with partial disability [42].

While these emerging successes are promising, barriers and future modification to social robot exercise program delivery will need to be addressed prior to broad reaching dissemination and adoption of this exercise delivery mode by older adults and healthcare providers. Among the barriers are the limitations of contemporary social robot technology to fully match the spontaneity and unpredictable nature of human movement velocity and communication patterns. These incongruences are most pronounced when compensations to movement patterns brought about by osteo- and arthrokinematic changes of aging [43] or neurosensory deficits such as hearing loss are present [44]. Even in the absence of age-related biomechanical joint changes, reproducibility of angles and degrees of movement in a robot joint as compared to constructs of a human joint (e.g., ball-and-socket, hinge, pivot, ellipsoidal) may present as a barrier to an older adult attempting to imitate the movement patterns of a robot. Given that this study found some neutral and mixed results to the Almere Questionnaire and the internally developed robot perception questionnaire, it is possible these movement barriers were, in part, contributors to the older adults’ diverse reception.

Another key consideration identified in this study is the need for a pre-exercise assessment of safety prior to initiating exercise. Specifically, while all participants in the current study were community dwelling and self-reported being in generally good health, none of the participants had a resting BP measure categorized as “normal,” with a stage 2 hypertension reading most frequently observed among participants. Notably, one of the participants had an SBP reading that was identified by the healthcare professionals as unsafe to initiate exercise and required physician clearance and adjustments to medication dosing prior to participating in the exercise program.

While healthcare professionals are capable of making real-time clinical decisions related to exercise intervention safety, robotic interfaces adept at measuring vital signs and then using programmed algorithms for general or person-specific safety guidance are warranted. Current telehealth monitoring technologies present viable opportunities for robotic monitoring of vital signs before, during, and after exercise to assure the safety of and compliance with the established exercise parameters. Inclusion of this feature within the capacity of social robots’ provides adjunctive healthcare value to delivering clinically acceptable, evidence-based healthcare exercise programs. An additional safety consideration when delivering standing exercises to older adult populations is fall risk mitigation. Investigators recognized the ethics board requirement of this study necessitated donning of a safety belt and providing safety personnel in close proximity to reduce participant fall risk does introduce human bias. The enforced need for fall avoidance measures during exercise is evidence of the widespread concern of a fall event in this population. The need for additional safety personnel does present an ethical dilemma as it is routine clinical practice for physical therapists to provide a written home exercise program or videos to complement in-clinic physical therapy or after conclusion of physical therapy. However, in routine physical therapist practice, several sessions of education on proper performance is often required before providing a home exercise program, especially with those at high risk of falls. As a future goal of robot assistance with exercise performance is the lack of frequent and direct human involvement, future studies will have to carefully navigate the absence or presence of direct human involvement with participants while still assuring safety and harm prevention during interventions, especially as it was noted that some participants tended to gravitate toward interacting with the key personnel instead of the robot. One way to reduce this need for safety personnel is to have a trained clinician teach the participant the exercise, and when they are proficient in performance, transition to robotic facilitation of exercise would be warranted as would occur with a physical therapist-prescribed home exercise program.

A prior study reported outcomes of a robotic balance training program and suggests that it may achieve the same effect as conventional balance training on postural stability in patients with a diagnosis of PD [13]. However, it is important to recognize that the study by Spina et al. excluded individuals with balance impairments due to non-PD conditions, and it is not clear what specific safety precautions or human interactions were employed within the study protocol to minimize the individual’s fall risk. Therefore, generalized recommendations of best practice fall reduction strategies for older adults participating in robot-led exercise cannot be generated but should be considered an important safety aspect of future protocol designs.

While inferential statistics are not available for the outcomes of this cohort study given the small population sample, positive trends were identified in the following outcomes: ABC scale, TUG, gait speed, and the SPPB following the conclusion of the program. Specifically, MCID was identified in the TUG and SPPB scores of some of the participants, and while no MCID values have been published for gait speed, substantial improvements to participants’ gait speed were identified as defined by Kwok et al. [37]. As the exercise program in the current study only measured change over a four-week timeframe, it is feasible that further outcome improvements could be made had the exercise protocol continued over a longer duration and warrants further examination. The investigators also recognize that the OEP Level B may not be an optimal exercise prescription for high-functioning participants compared to lower-functioning individuals. It is therefore possible that the OEP Level B exercise prescription dosing for study participants with relatively high-functioning baseline outcome measures may have had a ceiling effect and may not have fully realized the benefit of robot-led exercise. The diverse group of age and ability levels of the participants led to knowledge translation, executive functioning, and exercise performance at varying rates and ability levels. The robot was unable to accommodate adaptation to instructional speed due to the uniform programming and therefore may not have met the unique needs of each participant. In future studies, it would be beneficial to have various levels of exercise to best match the skill and fitness level of each participant. Specific to this study protocol, developing NAO robot programming capable of providing all four OEP exercise levels (A, B, C, and D) and a treadmill or bike option for individuals whose gait speed outpaces the Pepper robot walking speed is suggested. Finally, the investigators anecdotally identified this robot-led exercise protocol to be most effective for low- and moderate-functioning participants. However, this assertion requires further examination.

Study limitations

The small sample size was referred from one community center with varying comorbidities; therefore, the findings cannot be generalized broadly to all older adults. Additionally, human interaction required for safety and robot behavior limitations may have introduced bias to the study outcomes. Finally, despite emerging advancements in social robots’ capabilities, fully reproducible human movement mechanics and communication fluidity that match the expectations of older adults are not yet available.

Future research

Future research related to the use of robot-assisted exercise programs in an older adult population is expansive. Related to the findings of this study, the next steps for research include protocols designed to further reduce the human element of the protocol while still ensuring the safety of exercise participation. This may include adding a vital sign measurement interface with a decision-making algorithm that can provide caution to exercise as appropriate. Additionally, the use of the Pepper robot and its VR feature to guide aerobic exercise that has a reduced fall risk (i.e., stationary biking, elliptical) may limit the potential for human bias in future study iterations. Finally, the use of robot-assisted home exercise programs may be best studied in the home of an older adult and should be considered in future study designs.

## Conclusions

Robots have the potential for use as a supplement to physical therapist-prescribed home exercise programs and/or real-time exercise partners for community-validated independent prevention-focused programs. While ongoing development of robotic systems capable of delivering evidence-based physical therapy programming is warranted, this study provides an initial framework for validating the health efficacy of robot-facilitated exercise interventions as a complement to physical therapist services in the future.

## Appendices

Appendix 1.

**Table 8:** Perceptions of the Robotic Exercise Questionnaire

Perception of Effectiveness
1. Exercising with a robot is more or less effective than exercising by yourself?
I have not exercised by myself
Much more effective
More effective
About the same
Less effective
Much less effective
2. Exercising with a robot is more or less effective than exercising with a group of people?
I have not exercised by myself
Much more effective
More effective
About the same
Less effective
Much less effective
3. Exercising with a robot is more or less effective than exercising with an exercise video?
I have not exercised by myself
Much more effective
More effective
About the same
Less effective
Much less effective
Perceptions of Motivation
4. I am more or less motivated to exercise with a robot as compared to exercising by yourself?
I have not exercised by myself
Much more motivated
More motivated
About the same
Less motivated
Much less motivated
5. I am more or less motivated to exercise with a robot as compared to exercising with a group of people?
I have not exercised by myself
Much more motivated
More motivated
About the same
Less motivated
Much less motivated
6. I am are more or less motivated to exercise with a robot as compared to exercising with a video?
I have not exercised by myself
Much more motivated
More motivated
About the same
Less motivated
Much less motivated
Overall Perceptions
7. It will be (or is) difficult to follow along with exercises demonstrated by a robot
Strongly agree
Agree
Neutral
Disagree
Strongly disagree
8. Exercising with a robot is more preferred to exercising with a person
Strongly agree
Agree
Neutral
Disagree
Strongly disagree
9. Exercising with a robot improves my access to exercise
Strongly agree
Agree
Neutral
Disagree
Strongly disagree

Appendix 2.

**Table 9:** Modified Almere Questionnaire Please select the most appropriate level of agreement pertaining to your current status. 1 = Strongly disagree 2 = Disagree 3 = Neutral 4 = Agree 5 = Strongly agree

Question	Likert Scale Rating
If I should use the robot, I would be afraid to make mistakes with it	1 2 3 4 5
If I should use the robot, I would be afraid to break something	1 2 3 4 5
I find the robot scary	1 2 3 4 5
I find the robot intimidating	1 2 3 4 5
I think it’s a good idea to use the robot	1 2 3 4 5
The robot would make life more interesting	1 2 3 4 5
It’s good to make use of the robot	1 2 3 4 5
I think the robot can be adaptive to what I need	1 2 3 4 5
I think the robot will only do what I need at that particular moment	1 2 3 4 5
I think the robot will help me when I consider it to be necessary	1 2 3 4 5
I enjoy the robot talking to me	1 2 3 4 5
I enjoy doing things with the robot	1 2 3 4 5
I find the robot enjoyable	1 2 3 4 5
I find the robot fascinating	1 2 3 4 5
I find the robot boring	1 2 3 4 5
I consider the robot a pleasant conversational partner	1 2 3 4 5
I find the robot pleasant to interact with	1 2 3 4 5
I feel the robot understands me	1 2 3 4 5
I think the robot is nice	1 2 3 4 5
I think the robot is useful to me	1 2 3 4 5
It would be convenient for me to have the robot	1 2 3 4 5
I think the robot can help me with many things	1 2 3 4 5
When interacting with the robot I felt like I’m talking to a real person	1 2 3 4 5
It sometimes felt as if the robot was really looking at me	1 2 3 4 5
I can imagine the robot to be a living creature	1 2 3 4 5
I often think the robot is not a real person	1 2 3 4 5
Sometimes the robot seems to have real feelings	1 2 3 4 5
I would trust the robot if it gave me advice	1 2 3 4 5
I would follow the advice the robot gives me	1 2 3 4 5
